# Applicant Personality and Procedural Justice Perceptions of Group Selection Interviews

**DOI:** 10.1007/s10869-015-9430-9

**Published:** 2015-12-23

**Authors:** Hege H. Bye, Gro M. Sandal

**Affiliations:** Department of Psychosocial Science, University of Bergen, P.O. Box 7807, 5020 Bergen, Norway

**Keywords:** Group selection interview, Personality, Applicant reactions, Procedural justice, Fairness perceptions, Five-factor model, Statistical interaction

## Abstract

**Purpose:**

We investigated how job applicants’ personalities influence perceptions of the structural and social procedural justice of group selection interviews (i.e., a group of several applicants being evaluated simultaneously). We especially addressed trait interactions between neuroticism and extraversion (the affective plane) and extraversion and agreeableness (the interpersonal plane).

**Design/Methodology/Approach:**

Data on personality (pre-interview) and justice perceptions (post-interview) were collected in a field study among job applicants (*N* = 97) attending group selection interviews for positions as teachers in a Norwegian high school.

**Findings:**

Interaction effects in hierarchical regression analyses showed that perceptions of social and structural justice increased with levels of extraversion among high scorers on neuroticism. Among emotionally stable applicants, however, being introverted or extraverted did not matter to justice perceptions. Extraversion did not impact on the perception of social justice for applicants low in agreeableness. Agreeable applicants, however, experienced the group interview as more socially fair when they were also extraverted.

**Implications:**

The impact of applicant personality on justice perceptions may be underestimated if traits interactions are not considered. Procedural fairness ratings for the group selection interview were high, contrary to the negative reactions predicted by other researchers. There was no indication that applicants with desirable traits (i.e., traits predictive of job performance) reacted negatively to this selection tool.

**Originality/Value:**

Despite the widespread use of interviews in selection, previous studies of applicant personality and fairness reactions have not included interviews. The study demonstrates the importance of previously ignored trait interactions in understanding applicant reactions.

## Introduction

Perceptions of organizational justice are central antecedents of important outcomes in organizations, such as task performance, job satisfaction, citizenship behavior, and counterproductive work behaviors (Cohen-Charash and Spector 2001; Colquitt et al. 2001, 2013; Viswesvaran and Ones 2002). In part, perceived justice influences work outcomes through people’s perceptions of the quality of their social exchanges at work and the negative and positive affect triggered by (un)fair events or circumstances (Colquitt et al. 2013). People form judgments of justice quickly and the perceived fairness of a single event, such as a selection interview, may be sufficient to influence subsequent work-related behaviors (Colquitt et al. 2013; Lind 2001). In selection contexts, applicants’ perceptions of the fairness of the hiring process have been shown to influence their attraction to the organization, intentions to recommend the employer to others, and intentions to accept job offers (Hausknecht et al. 2004), as well as actual job choice through effects on acceptance intentions (Chapman et al. 2005). Moreover, there is some evidence that applicants’ reactions are indirectly related to job performance by influencing test scores in selection (McCarthy et al. 2013).

Against this backdrop, employers could benefit from trying to affect applicants’ reactions in selection. This may be achieved through the design and implementation of selection systems (Truxillo and Bauer 2011), but it may also prove important to take into account individual differences within the applicant pool. Research demonstrates substantial variability in applicants’ reactions to selection tools, suggesting that applicants’ reactions are not only shaped by external factors associated with the specific selection context (i.e., factors under the organization’s control), but are also shaped by individual differences (Ryan and Huth 2008). For selection professionals, it may be useful to know how much of the variation in applicant reactions is bound by the applicants’ dispositions and thus not amenable to change by the organization (Honkaniemi et al. 2013; Truxillo et al. 2006). Moreover, understanding which specific traits shape applicants’ reactions can be useful in selection system design. If one has knowledge of dominant traits in the candidate pool, some selection tools can be chosen or avoided (Merkulova et al. 2014; Ryan and Huth 2008; Truxillo et al. 2006).

Models of applicant reactions do include personality factors as proposed antecedents of justice perceptions (Hausknecht et al. 2004; Ryan and Ployhart 2000). However, there are still few studies that have examined applicant personality in relation to fairness reactions (Hausknecht et al. 2004; Truxillo and Bauer 2011) and the extant research has some notable limitations. First of all, previous studies of applicant personality and fairness reactions have not focused explicitly on interviews. This is surprising considering the widespread use of interviews in selection (Salgado et al. 2001). Applicant personality traits and selection methods may interact so that relationships between traits and reactions do not generalize from one selection tool to another (Merkulova et al. 2014; Oostrom et al. 2010). It is therefore important to study relationships between applicant personality and fairness reactions also in the context of interviews.

Secondly, extant studies of personality and applicant fairness perceptions have largely investigated bivariate relationships or additive effects of personality traits (Bernerth et al. 2006; McFarland 2003; Merkulova et al. 2014; Oostrom et al. 2010; Truxillo et al. 2006), which leaves unaddressed the potentially important effects of specific interactions between traits (Honkaniemi et al. 2013). Finally, quite a few studies in this area have employed student samples in classroom or lab-settings (Bernerth et al. 2006; McFarland 2003; Oostrom et al. 2010; Wiechmann and Ryan 2003). Because there is evidence to suggest that justice is weighted more heavily among actual applicants (Chapman et al. 2005), it is important to complement findings from the lab with studies in the field.

We address these issues by conducting a field study examining how applicants’ personality traits, including two theoretically important trait interactions, influence how group selection interviews are experienced in terms of procedural fairness. We begin by providing a brief description of group selection interviews.

### Group Selection Interviews

Assessing candidates in groups has a long history in personnel selection (Ansbacher 1951) and remain popular either as part of assessment centers (Krause and Thornton 2009) or alone. Group interviews involve the interviewing of a group of applicants by one or more interviewers/assessors, and should not be confused with panel interviews in which a group of interviewers evaluate a single applicant. Unlike leaderless group discussions, in which candidates are given one or more issues to discuss without additional prompts, group selection interviews can be more structured and may involve presentation of individual applicants, issues for discussion, and specific questions from the interviewer(s). Organizations may choose to implement group interviews for several reasons. A complete assessment center may be considered too resource demanding (Shechtman 1991) and group interviews are cost-effective in situations where many applicants need to be assessed in a short period of time. Group interviews may also be seen as facilitating the comparison of applicants for the same or similar positions, and as a good tool to assess candidates’ interaction skills (Tran and Blackman 2006).

Research from the field of education, where group interviews have been employed in the selection of students into teacher education programs, shows that group interviews can be reliable and predictive of performance (Byrnes et al. 2003; Faulk 2008; Shechtman 1991, 1992; Shechtman and Sansbury 1989). Beyond this research, very little information on the group interview is available for practitioners (Leshem 2012). Concerns have been raised that applicants may experience group interviews as unfair, as the group setting may compromise privacy and allow for less individual consideration (Tran and Blackman 2006). Knowing whether these concerns are warranted is clearly important for employers who use, or consider using, group interviews in selection.

### Applicant Personality and Perceptions of Procedural Justice

Research on applicant reactions to selection procedures commonly draw on the perspective of organizational justice (Greenberg 1990) and focus on distributive justice (fairness of outcomes) and procedural justice (fairness of the procedures employed in decision making) (Gilliland 1993). We focus on procedural justice, as our field study design did not allow for data collection after the selection outcome was known to the applicants. Gilliland (1993) proposed that the satisfaction or violation of several justice rules (e.g., job-relatedness, selection information, sufficient two-way communication) underlie applicants’ overall perceptions of procedural justice in selection. Later, Bauer et al. (2001) showed that these justice rules could be seen as underlying perceptions of the structural and social procedural justice of a selection tool. *Structural aspects* concern perceptions that the test is job-related, provides an opportunity to show one’s skills, and that information given about the test is adequate. *Social aspects* concern perceptions that all applicants are treated similarly and in an open and polite manner, that questions are not prejudiced or too personal, and that there is sufficient two-way communication during the testing process. This structure fairness/social fairness framework is commonly used in applicant reactions research (Truxillo et al. 2009).

Among the individual differences that may impact on applicants’ perceived structural and social fairness, we focus on the personality dimensions described in the five-factor model of personality: neuroticism, extraversion, agreeableness, openness-to-experience, and conscientiousness (Costa and McCrae 1992a; McCrae and Costa 1987). These traits are related to important outcomes at work, such as performance, motivation satisfaction, organizational citizenship behaviors, and general perceptions of organizational justice (Chiaburu et al. 2011; Judge et al. 2002; Judge and Ilies 2002; Salgado 1997; Shi et al. 2009). Importantly, a focus on these five traits allows for comparisons of our results with studies of reactions to other selection tools in which all or some of the five traits were included (Bernerth et al. 2006; McFarland 2003; Merkulova et al. 2014; Oostrom et al. 2010; Truxillo et al. 2006; Van Vianen et al. 2004; Wiechmann and Ryan 2003).

There is a growing awareness in research on personality traits and work behaviors that traits interact to influence important outcomes, beyond any “main effects” of the individual traits (Burns et al. 2014; Jensen and Patel 2011; Judge and Erez 2007; Witt et al. 2002). Apart from Honkaniemi et al.’s (2013) study of personality types and applicant reactions, this issue has been overlooked in previous studies of personality traits and applicant reactions. We therefore focus on two trait combinations which should be especially relevant to how individuals experience the group interview: the interaction between neuroticism and extraversion (the affective plane) and the interaction between extraversion and agreeableness (the interpersonal plane) (Costa and McCrae 1992b).

Unlike other common selection tools such as written or computerized tests, group interviews have a very strong social component. They combine the interpersonal aspects of one-on-one interviews with the group dynamics of interacting with the other applicants and the interviewer(s)/assessor(s). Given these strong social features of group interviews as a selection tool, the interpersonal plane of personality (i.e., the E × A interaction) should be important to applicants’ experiences and reactions, as we argue in more detail below.

Moreover, group interviews combine intense social interaction with the purpose of evaluation inherent to all selection tools. The applicants are not just being evaluated by the interviewer; they are evaluated in front of, and indirectly also by, the other applicants. Thus, group interviews are likely to represent a social-evaluative threat (Dickerson and Kemeny 2004). Humans are motivated to preserve their social selves: their social esteem, status, and acceptance. Social situations in which poor performance is likely to be seen as reflecting the lack of a desired trait or ability constitute a threat to this goal (Dickerson and Kemeny 2004). How individuals react to social-evaluative threats is in part modulated by their personality, especially traits that relate to the experience of negative and positive affect (Childs et al. 2014). This provides a theoretical rationale for focusing on the affective plane (i.e., the N × E interaction) in reactions to group interviews.

#### Neuroticism and Extraversion

Together, neuroticism and extraversion represent individuals’ basic emotional styles. Consistent with research demonstrating the important role of trait negative affect and trait positive affect in the general organizational justice literature (Barsky and Kaplan 2007), we expect these traits to influence candidates’ experiences of the interview as (un)fair. High scorers on neuroticism tend to be insecure, worrying, and self-conscious. Low scorers are characterized as emotionally stable; relaxed, comfortable, and hardy. Extroverted individuals tend to be sociable, talkative, active, and person-oriented, whereas introverted individuals tend to be more reserved and inhibited (McCrae and Costa 1987). A higher level of neuroticism is associated with more negative affect and a higher level of extraversion is associated with more positive affect. Importantly, introversion does not dictate a presence of negative affect, but simply a lesser tendency to experience positive affect. Similarly, emotional stability is primarily associated with the absence of negative affect, not the presence of positive affect (Costa and McCrae 1980, 1992b).

Results from previous studies investigating the main effects of neuroticism on fairness perceptions are mixed. In their meta-analysis, Hausknecht et al. (2004) found a mean sample-weighted correlation of −0.04 between neuroticism and applicant perceptions of procedural justice. In a study among students who completed a cognitive ability test and a multimedia situational judgment test (SJT), Oostrom et al. (2010) found that higher levels of emotional stability correlated positively with perceived face and predictive validity of the cognitive ability test. Perceived face validity and perceived predictive validity both concern job-relatedness and are aspects of structural fairness. Other field studies, however, have failed to find a significant correlation between aspects of structural fairness and neuroticism or negative affectivity (Bauer et al. 2001; Merkulova et al. 2014; Truxillo et al. 2006).

Regarding social justice, Truxillo et al. (2006) found that neuroticism was negatively correlated with the perceived social fairness of a multiple choice test. Similarly, Bauer et al. (2001) found that negative affectivity was negatively correlated with the perceived social fairness of a cognitive ability test, but it was not related to the perceived social fairness of participants’ last interview experience. Taken together, these studies provide some indication that neuroticism is negatively related to both structural and social justice.

Hausknecht et al. (2004) did not report meta-analytic findings regarding extraversion and perceived procedural justice. Later research has also failed to find significant correlations between extraversion and perceived social or structural fairness (Merkulova et al. 2014; Oostrom et al. 2010; Truxillo et al. 2006). We suggest that the effect of extraversion on justice perceptions of group interviews is conditioned on the applicants’ standing on the neuroticism dimension. As suggested by Truxillo et al. (2006), interviews may be less attractive to introverted than extraverted applicants. However, this may hold true only for those introverts who are also low in emotional stability. At higher level of emotional stability, levels of extraversion may be less predictive of fairness perceptions; being relaxed, comfortable, and hardy may be enough to render the group interview a positive experience even for applicants who are not particularly outgoing or talkative.

Support for this line of reasoning can be found in the study by Honkaniemi et al. (2013) on personality types. They demonstrated that applicants with an overcontrolled personality profile (including high scores on neuroticism and low scores on extraversion) rated a selection process as less fair than applicants who were characterized as resilient (including low scores on neuroticism and high scores on extroversion) when controlling for the effects of the individual big five traits. Against this backdrop, we predict that

##### **Hypothesis 1**

Applicants’ levels of neuroticism and extraversion interact in the prediction of perceived justice. Specifically, the negative effect of higher neuroticism on (a) social procedural justice and (b) structural procedural justice will be stronger for applicants who are low on extraversion (i.e., introverted).

#### Agreeableness and Extraversion

Together, agreeableness and extraversion define the interpersonal plane (Costa and McCrae 1992b; McCrae and Costa 1989) and these traits may interact to shape applicants’ trust and comfort in the interaction with other candidates and the interviewer. High scorers on agreeableness tend to be flexible, sympathetic, trusting, and generous. Low scorers on the other hand tend to be mistrustful, skeptical, and uncooperative (McCrae and Costa 1987). Merkulova et al. (2014) found that agreeable applicants rated an assessment center as higher on face validity and measurement quality (i.e., structural fairness). Oostrom et al. (2010) found that agreeableness was positively related to aspects of perceived structural fairness of a cognitive ability test, but not a SJT. Conversely, Truxillo et al. (2006) found that agreeableness was positively correlated with the social, but not structural, fairness of a multiple choice test. Agreeableness has also been shown to predict the perceived overall procedural justice of a personality test (Bernerth et al. 2006).

The tendency to be trusting and flexible in interpersonal relationships should lead agreeable applicants to experience the group interview setting more positively in terms of both social and structural fairness. Moreover, the positive effect of high agreeableness may be strengthened by higher levels of extraversion; a candidate who is both agreeable and extraverted (outgoing, assertive) may be especially likely to evaluate a group interview setting as fair. At lower levels of agreeableness, however, extraversion may not contribute much to perceived fairness; being argumentative, skeptical, and uncooperative may predispose applicants to view groups interviews as less fair, regardless of their standing on the extraversion dimension. In line with this argument, Honkaniemi et al. (2013) found that resilient (including high extraversion and high agreeableness) applicants expressed more positive fairness reactions than overcontrolled (including low extraversion and low agreeableness) applicants.

##### **Hypothesis 2**

Applicants’ levels of agreeableness and extraversion interact in the prediction of perceived justice. Specifically, ratings of (a) social procedural justice and (b) structural procedural justice will be highest for applicants with high scores on both agreeableness and extraversion.

#### Openness-to-Experience

In addition to the two trait interactions described above, we also expect openness-to-experience to predict applicants’ fairness perceptions. Open individuals are characterized by being original, imaginative, and creative. More closed individuals tend to be conventional and conservative, and prefer familiar rather than novel experiences (Costa and McCrae 1992b; McCrae and Costa 1987). Ryan and Ployhart (2000) suggested that open individuals may be more positive to innovative selection procedures. It may also be the case that open applicants are generally more positive to selection tools because they may involve intellectually challenging and novel tasks (e.g., ability tests), require imagination in the consideration of hypothetical scenarios (e.g., situational interviews, SJTs), or offer the opportunity to discuss complex professional issues (e.g., interviews).

Oostrom et al. (2010) found that openness-to-experience positively correlated with the perceived face and predictive validity of a cognitive ability test and the face validity of a multimedia SJT. Similarly, openness has been found to be positively correlated with the perceived face validity of a computerized in-basket examination (Wiechmann and Ryan 2003). This suggests that openness is related to applicants’ perceptions of structural fairness.

There is also evidence that openness is positively related to perceived social fairness of a multiple choice test (Truxillo et al. 2006). Moreover, openness has been found to correlate positively with the overall perceived procedural justice a personality test (Bernerth et al. 2006) and to indirectly affect the general perceived fairness of a selection process consisting of personality, cognitive ability, and situational judgment tests (Van Vianen et al. 2004). Against this backdrop, we propose that applicants’ levels of openness shape their perceptions of both the social and structural fairness of the group interview.

##### **Hypothesis 3**

Applicants’ scores on openness-to-experience positively predict ratings of (a) social procedural justice and (b) structural procedural justice.

#### Conscientiousness

Conscientious individuals are careful, reliable, and hardworking. They also tend to be ambitious and energetic (McCrae and Costa 1987). Hausknecht et al. (2004) found a mean sample-weighted correlation of 0.08 between conscientiousness and applicant perceptions of procedural justice. Consistent with this finding, results from later research are mixed regarding relationships between conscientiousness and fairness perceptions (Merkulova et al. 2014; Oostrom et al. 2010; Truxillo et al. 2006). We do not propose that applicants’ level of conscientiousness is related to their fairness perceptions of group selection interviews. However, we include this dimension for completeness and exploratory purposes.

## Method

### Sample and Procedure

Our data come from a selection process for teacher positions at a Norwegian public high school. Teachers were hired in 2010, 2011, and 2012. The selection procedure was developed by the school leaders. Applications and CVs were evaluated before eligible candidates were invited to group interviews. This screening focused on the applicants’ formal qualifications in the relevant areas of teaching, applicants’ previous experience teaching at the age/grade level that the school’s students would be at, applicants’ statements about their own views and values related to teaching, and applicants’ interest in and knowledge about the school’s teaching philosophy and priorities. Each interview lasted 2–3 h and involved three to five applicants. The assistant principal facilitated the interviews, whereas the principal and a union representative observed. First, applicants were informed about the process and agreed to an obligation of confidentiality. It was stressed that many positions were available and that several of the applicants could be hired. Next, the assistant principal presented the school and its teaching philosophy. Then, the applicants presented themselves, their background and motivation.

The next part was a structured dialogue among the applicants. A pool of statements was prepared by the school leaders prior to the interview. Each statement concerned questions and values related to teaching. The first applicant drew a statement from a bowl, read it out loud, and reflected on the statement. Next, each of the other applicants presented their reflections. The first applicant then summarized the group’s viewpoints, and the other applicants were allowed to change or add to their original comments. The process was repeated several times, alternating positions between the applicants. Many of the statements remained constant across the 3 years; a few were changed or added. Importantly, all statements were written to elicit applicants’ responses relevant to the evaluation criteria (e.g., views on students and learning). The criteria remained the same across the 3 years.

After a short break, the applicants and the assistant principal discussed pedagogical challenges, views on students and learning, attitudes to innovation and the use of information technology. Finally, applicants were invited to ask questions and the assistant principal and the observers asked more specific follow-up questions to the applicants.

When the school invited applicants to interviews, they also sent out invitations to the study and a link to a questionnaire with personality measures (time 1). The confidentiality of their responses was stressed and it was made explicit that the questionnaire data (e.g., the personality measures) would not be made available to the school leaders and were not part of the selection process. Applicants were instructed to fill in the questionnaire prior to taking part in the interview. After the interviews, but before the section decisions were made known to the applicants, the school sent out a link to a second questionnaire measuring fairness perceptions with respect to the interview (time 2). Again, confidentiality was stressed. The two questionnaires were matched based on the applicants’ date of birth. This procedure was approved by the Norwegian Data Protection Official for Research.

In total, 129 applicants responded at time 1 and 106 responded at time 2. Some responded only at time 1 (*n* = 32) or time 2 (*n* = 9). The final sample consisted of 97 applicants. The overall response rate across the 3 years (i.e., 2010, 2011, and 2012) could not be calculated due to a practical error in the 2012 data collection. However, in the 2010 and 2011 data collection the combined response rate was 89.7 % (93.9 and 84.3 %, respectively). From time 1 to time 2, 75.2 % of the respondents remained in the study. Among the 97 participants, 54 were women and the majority was between 36 and 45 years old (37.2 %).

### Measures

#### Personality

Applicants’ personality traits were assessed by the Norwegian translation of the NEO-Five-Factor Inventory (NEO-FFI; Costa and McCrae 1992b; Martinsen et al. 2005). Each of the five dimensions is measured by 12 items on a five-point scale from *strongly disagree* (0) to *strongly agree* (4). Scores across the 12 items are summed after reversing negatively formulated items. Cronbach’s alphas ranged from 0.72 for openness-to-experience to 0.82 for neuroticism.

#### Procedural Justice

We could not identify an established scale to measure fairness perceptions of group interviews, necessitating the development of a new scale. Based Gilliland’s (1993) theoretical work on the perceived fairness of selection systems and the work by Bauer et al. (2001), a 20-item questionnaire assessing applicants’ reactions to the group interview was developed. Some items were adapted from Bauer et al.’s (2001) scale to measure fairness perceptions of selection tests and some were tailored to the group interview setting. Eleven items measured social fairness (e.g., “I was treated with consideration and respect during the group interview”). Nine items measured structural fairness (e.g., “I had the opportunity to demonstrate my competence during the interview”). All items are listed in Table 1. Cronbach’s alphas for the social and structural fairness scales were 0.85 and 0.81, respectively. Due to the modest size of the sample, exploratory or confirmatory factor analyses could not be performed.

**Table 1:** Questionnaire items employed to measure structural and social justice

Structural justice items	Social justice items
I experienced the group interview as relevant for the job	Everyone was treated equally in the group interview^a^
The group interview covered topics that are important for the job	Everyone had the same opportunity to show they can do and what they stand for
I believe that the information that came out of the group interview provides a sound foundation for the hiring decision	I experienced the interviewer as honest and sincere^a^
I had the opportunity to demonstrate my competence during the interview^a^	I was treated with consideration and respect during the group interview^a^
I had the opportunity to present my input and viewpoints in important areas	I was given the chance to provide input during the process
The participants were given the same opportunities and were treated fairly	I was given the chance to ask questions about the position
I was well informed in advance about what the group interview would entail^a^	I was given the chance to ask questions about the work place
I knew what I could expect when I arrived at the interview^a^	I was given the chance to ask questions about the hiring process
The interviewer gave a thorough description of the process at the beginning of the interview	None of the questions in the interview were offensive
	None of the questions in the interview were too personal^a^
	None of the questions in the interview appeared prejudiced^a^

^a^Adapted from Bauer et al. (2001)

The social fairness scale (*M* = 4.53, *SD* = 0.54) exhibited significant negative skewness (skewness = −1.868, *SE* = 0.245, *z* = −7.62, *p* < 0.001) and significant kurtosis (kurtosis = 4.734, *SE* = 0.485, *z* = 9.76, *p* < 0.001). To remedy this, the social fairness variable was transformed (i.e., reflected and inversed) to approach a normal distribution. Similarly, the structural fairness variable (*M* = 4.19, *SD* = 0.58) also exhibited significant skewness (skewness = −0.803, *SE* = 0.245, *z* = −3.28, *p* < 0.001) and was transformed (i.e., reflected and inversed). Our choice of transformations was based on the recommendations by Tabachnick and Fidell (2007). They write

If the distribution differs severely the inverse is tried. According to Bradley (1982), the inverse is the best of several alternatives for J-shaped distributions …

The direction of the deviation is also considered. … If there is negative skewness, the best strategy is to *reflect* the variable and then apply the appropriate transformations for positive skewness^16^. To reflect a variable, find the largest score in the distribution and add one to it to form a constant that is larger than any score in the distribution. Then create a new variable by subtracting each score from the constant. In this way, a variable with negative skewness is converted to one with positive skewness prior to transformation. (Tabachnick and Fidell 2007, p. 88).

To find the inverse (NEW X) of a variable (X), divide 1 by the variable scores. In SPSS syntax: NEWX = 1/X (see Table 4.3 in Tabachnick and Fidell 2007, p. 89)

The transformed variables showed a more normal distribution: structural fairness (skewness = 0.278, *SE* = 0.245, *z* = 1.13, *ns.*; kurtosis = −0.710, *SE* = 0.485, *z* = −1.46, *ns.*) and social fairness (skewness = −0.366, *SE* = 0.245, *z* = −1.53, *ns*; kurtosis = −0.993, *SE* = 0.485, *z* = 2.05, *p* < 0.05). The correlations between the raw and transformed scores were *r* = 0.93 for social fairness, and *r* = 0.95 for structure fairness.

## Results

Means, standard deviations and intercorrelations are presented in Table 2. To test our hypotheses, we performed hierarchical linear regression analyses with social and structural fairness as the outcome variables. In the prediction of social fairness, we entered applicants’ scores on the five traits in the first step. In the second step, we added the interaction terms. In the prediction of structural fairness, we also included applicant age as a control variable due to the significant correlation between these two variables. All predictors were centered on their means. The complete results are presented in Tables 3 and 4. Because our main focus was on the interaction effects, we estimated 95 % confidence intervals around their contribution to the explained variance (i.e., the *R* ^2^ change) in each of the two regression models. These calculations were based on the description by Smithson (2003, pp. 55–57) and his SPSS files for noncentral confidence interval calculations (Smithson 2015).

**Table 2:** Means, standard deviations, and intercorrelations among study variables

	*M*	*SD*	1	2	3	4	5	6	7	8
1. Sex^a^	–	–	–							
2. Age^b^	4.33	1.84	0.30**	–						
3. Neuroticism	13.49	6.76	−0.10	−0.14	–					
4. Extraversion	34.18	5.26	0.33**	0.10	−0.37***	–				
5. Agreeableness	37.70	4.65	0.30**	0.09	−0.36***	0.49***	–			
6. Openness-to-experience	32.70	5.63	0.16	0.01	0.14	0.14	0.23*	–		
7. Conscientiousness	36.82	5.34	0.27**	0.13	−0.38***	0.47***	0.55***	0.02	–	
8. Social fairness (transformed)	0.75	0.21	0.15	0.12	−0.18^†^	0.30**	0.34***	0.05	0.22*	–
9. Structure fairness (transformed)	0.61	0.18	0.15	0.24*	−0.25*	0.29**	0.27**	0.00	0.15	0.53***

*** *p* < 0.001; ** *p* < 0.001; * *p* < 0.05; ^†^ *p* < 0.10

^a^Sex coded 0 = male, 1 = female

^b^Age categories scored from 1 (youngest) to 8 (oldest)

**Table 3:** Hierarchical regression analysis predicting perceptions of social fairness

Predictor	β	*t*	*p*	*R* ^2^	Adj. *R* ^2^	Δ*R* ^2^
Step 1						
Neuroticism	−0.02	−0.18	0.855	0.139	0.092	
Extraversion	0.18	1.51	0.134			
Agreeableness	0.25	1.97	0.052			
Openness	−0.03	−0.32	0.753			
Conscientiousness	−0.01	−0.06	0.949			
Step 2						
Neuroticism	0.04	0.38	0.705	0.199	0.136	0.060*
Extraversion	0.16	1.34	0.184			
Agreeableness	0.32	2.51	0.014			
Openness	−0.06	−0.57	0.573			
Conscientiousness	0.01	0.11	0.912			
N × E	0.21	2.09	0.039			
E × A	0.21	2.04	0.045			

* *p* < 0.05

**Table 4:** Hierarchical regression analysis predicting perceptions of structural fairness

Predictor	β	*t*	*p*	*R* ^2^	Adj. *R* ^2^	Δ*R* ^2^
Step 1						
Age	0.24	2.40	0.018	0.057	0.047	–
Step 2						
Age	0.20	2.08	0.040	0.167	0.112	0.110*
Neuroticism	−0.11	−1.02	0.308			
Extraversion	0.20	1.67	0.098			
Agreeableness	0.19	1.53	0.129			
Openness	−0.06	−0.55	0.584			
Conscientiousness	−0.12	−1.01	0.315			
Step 3						
Age	0.21	2.15	0.034	0.221	0.150	0.054
Neuroticism	−0.05	−0.48	0.629			
Extraversion	0.17	1.51	0.136			
Agreeableness	0.26	2.06	0.043			
Openness	−0.08	−0.79	0.431			
Conscientiousness	−0.10	−0.86	0.390			
N × E	0.20	1.98	0.050			
E × A	0.20	1.95	0.054			

* *p* < 0.05

In the prediction of social fairness, the overall regression model was significant in the first step, *F*(5, 91) = 2.94, *p* < 0.05, *R* ^2^ = 0.14. However, none of the effects of the five traits reached significance. The overall model including the two interaction terms in the second step was also significant, *F* (7, 89) = 3.16, *p* < 0.01, *R* ^2^ = 0.20, Δ*R* ^2^ = 0.06, *p* = 0.04, 95 % CI [0.00, 0.18]. The interaction of neuroticism and extroversion was significant, *β* = 0.21, *p* < 0.05, as was the interaction of extraversion and agreeableness, *β* = 0.21, *p* < 0.05, providing initial support for hypothesis 1a and hypothesis 2a. As openness-to-experience was not a significant predictor of social procedural justice, hypothesis 3a was not supported.

**Fig. 1:**
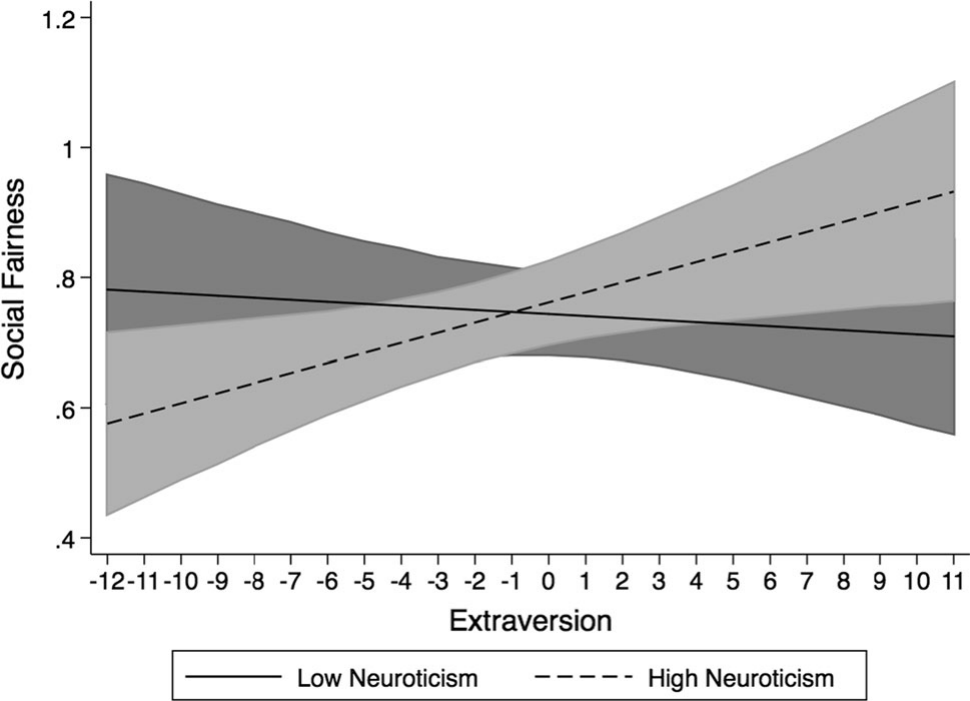
Interaction between extroversion and neuroticism in the prediction of perceived social fairness plotted at one standard deviation above and below the scale mean on neuroticism. *Shaded areas* represent the 95 % confidence intervals

The interaction of neuroticism and extroversion in the prediction of social fairness is plotted in Fig. 1. The simple slope of extroversion on social fairness at high levels of neuroticism was significant (*b* = 0.013, *t* = 2.05, *df* = 89, *p* < 0.05, two-tailed), suggesting that at high levels of neuroticism, applicants’ perceptions of social justice change with their level of extroversion so that emotionally unstable and introverted applicants perceive the interview as less socially fair than emotionally unstable and extroverted applicants. This supports hypothesis 1a. The simple slope of extroversion on social fairness at low levels of neuroticism, however, was not significant (*b* = −0.001, *t* = −0.12, *df* = 89, *p* > 0.05, two-tailed). This indicates that for applicants who are emotionally stable their level of extraversion does not impact on perceptions of social fairness.

**Fig. 2:**
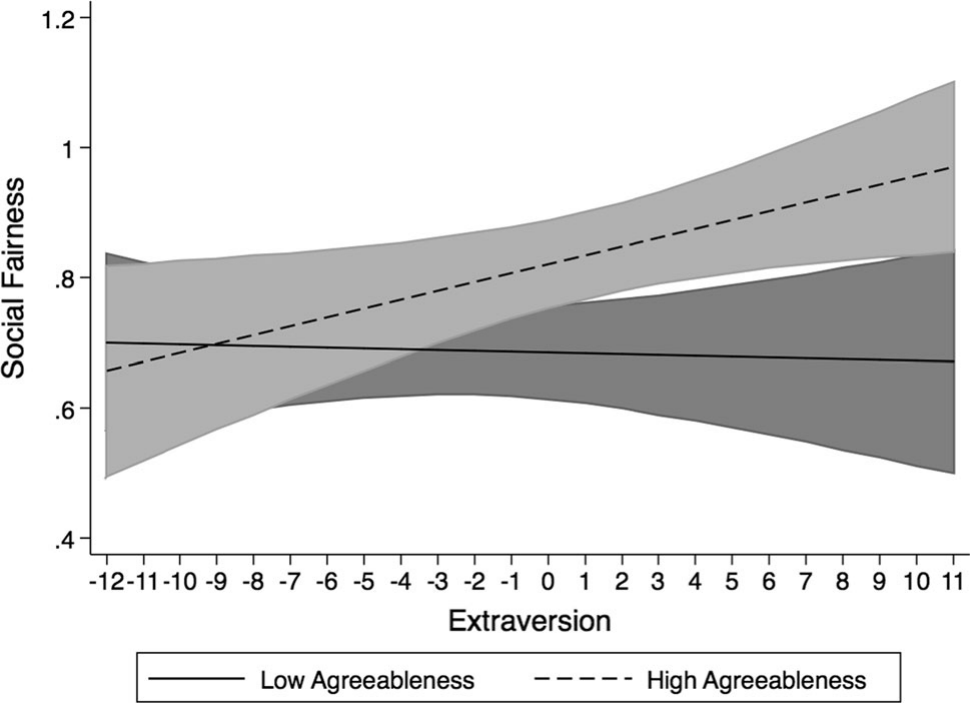
Interaction between extraversion and agreeableness in the prediction of social justice plotted at one standard deviation above and below the scale mean on agreeableness. *Shaded areas* represent the 95 % confidence intervals

The interaction of extraversion and agreeableness in the prediction of social justice is plotted in Fig. 2. The simple slope of extroversion on social fairness was not significant at low levels of agreeableness (*b* = −0.003, *t* = −0.54, *df* = 89, *p* > 0.05, two-tailed). However, at high levels of agreeableness, the simple slope of extraversion was significant (*b* = 0.015, *t* = 2.69, *df* = 89, *p* < 0.05, two-tailed). This indicates that for applicants who are low on agreeableness, their standing on the introversion–extroversion dimension is not important to their experience of social justice. Highly agreeable applicants, however, perceive the group interview as more socially fair as their level of extraversion increases. This supports hypothesis 2a.

In the prediction of structural justice, the first model containing only applicant age was significant, *F*(1, 95) = 5.77, *p* < 0.05, *R* ^2^ = 0.06. Adding the five traits also resulted in a significant model, *F*(6, 90) = 3.01, *p* = 0.01, *R* ^2^ = 0.17, Δ*R* ^2^ = 0.11, *p* = 0.045. However, none of the effects of the five traits reached significance. Adding the interaction terms also resulted in a significant model, *F*(8,88) = 3.12, *p* < 0.01, *R* ^2^ = 0.22, Δ*R* ^2^ = 0.05, *p* = 0.053, 95 % CI [0.00, 0.17]. In the third step, the interaction of neuroticism and extraversion was just significant *β* = 0.20, *p* = 0.05, as was the first level effect of agreeableness, *β* = 0.26, *p* < 0.05. This provided initial support for hypothesis 1b. The interaction between extraversion and agreeableness just missed significance (*p* = 0.054) and hypothesis 2b was therefore not supported. Hypothesis 3b predicting a significant effect of openness-to-experience on structural fairness was also not supported.

**Fig. 3:**
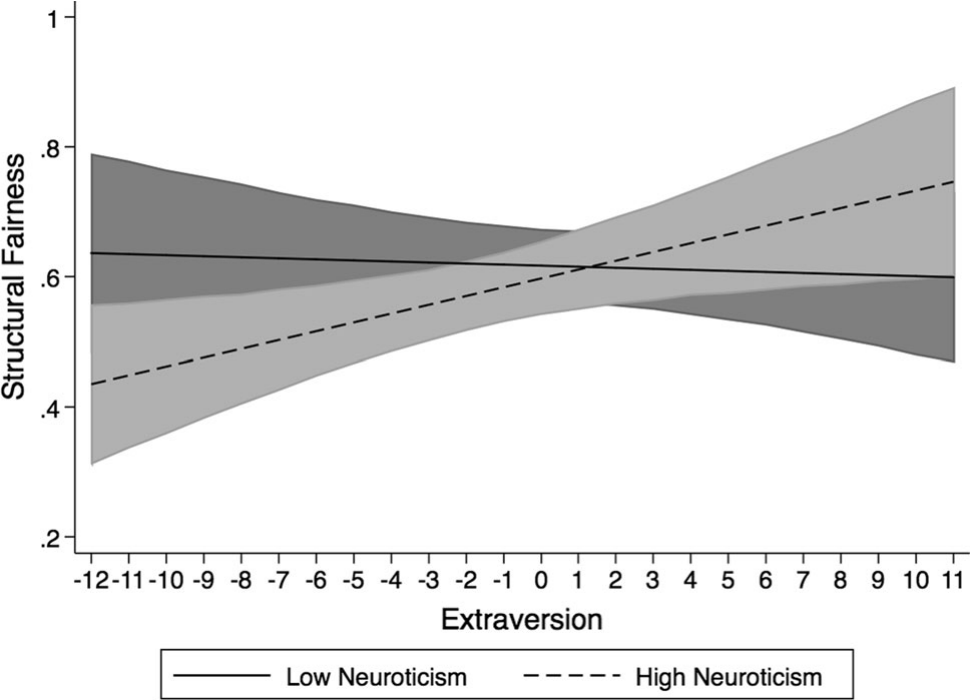
Interaction between neuroticism and extraversion in the prediction of structural justice plotted at one standard deviation above and below the scale mean on neuroticism. *Shaded areas* represent the 95 % confidence intervals

The interaction of neuroticism and extraversion in the prediction of structural fairness is plotted in Fig. 3. The simple slope of extroversion on structural fairness was not significant at low levels of neuroticism (*b* = −0.001, *t* = −0.12, *df* = 88, *p* > 0.05, two-tailed). However, at high levels of neuroticism, the simple slope of extraversion was significant (*b* = 0.013, *t* = 2.19, *df* = 88, *p* < 0.05, two-tailed). Thus, for emotionally stable applicants, levels of extroversion were not important to ratings of structural fairness. However, for applicants’ high in neuroticism, being introverted was associated with lower levels of perceived structural fairness. This supports hypothesis 1b.

## Discussion

The results showed that applicants’ traits explained an important part of the variance in perceptions of the fairness of group interviews. Applicants’ perceptions of social justice increased with levels of extraversion among high scorers on neuroticism. Among emotionally stable applicants, however, being introverted or extraverted did not matter to perceptions of social justice. Paralleling the findings for social justice, our results showed that among applicants high in neuroticism, being introverted was associated with lower levels of perceived structural fairness. Among emotionally stable applicants, levels of extroversion were not important to ratings of structural justice. Similarly, levels of extraversion did not impact on the perception of social justice for applicants low in agreeableness. Agreeable applicants, however, experienced the group interview as more socially fair when they were also extraverted. Contrary to our predictions, extroversion and agreeableness did not interact significantly in the prediction of structural fairness. Rather, there was a main effect of agreeableness; applicants who are trusting and flexible experienced the group interview as more structurally fair.

It is especially interesting to note that none of the five traits were significant predictors of fairness perceptions in the first steps of the regression analyses. Put differently, the conditional effects were not significant, but the incremental interactive effects were. Admittedly, the confidence intervals indicate that the variance in fairness perceptions explained by the interactions may range in size from essentially zero to medium/large. On the other hand, three out of four interaction effects were strong enough to reach significance, despite our modest sample size. Thus, the main contribution of the present study lies in the inclusion of the trait interactions and the finding that three out of four interaction effects were significant. Although our study is also limited in that we only considered two specific trait interactions (i.e., neuroticism x extraversion and extraversion x agreeableness), we believe that our results provide valuable nuances to the discussion on the impact of applicant personality on fairness perceptions. For example, contrary to the results in previous studies suggesting that extraversion is unrelated to fairness perceptions (Merkulova et al. 2014; Oostrom et al. 2010; Truxillo et al. 2006), our findings suggest that extraversion does relate to fairness perceptions but at specific levels of neuroticism and agreeableness. Our study also adds to the growing literature on how personality traits interact to shape individuals’ work-related behaviors more generally (Burns et al. 2014; Jensen and Patel 2011; Judge and Erez 2007; Witt et al. 2002).

Contrary to our hypotheses, openness-to-experience did not predict perceptions of fairness. This is inconsistent with previous research showing relationships between openness and aspects of the perceived fairness of cognitive ability, personality, situational judgment, multiple choice, and computerized in-basket tests (Bernerth et al. 2006; Oostrom et al. 2010; Truxillo et al. 2006; Van Vianen et al. 2004; Wiechmann and Ryan 2003). One possible explanation for the inconsistency is that openness-to-experience predicts the perceived fairness of individual-based and cognitively oriented testing, but not group-based, socially oriented testing. This interpretation is consistent with the results of Merkulova et al. (2014) who did not find a relationship between openness-to-experience and reactions to an assessment center consisting of group exercises, role-plays, and oral presentations.

Another, more technical explanation, concerns the measures employed in the studies. Openness-to-experience was significantly correlated with the four other traits (*r*s ranging from ±0.17 to 0.51) in the studies by Oostrom et al. (2010) and Truxillo et al. (2006). In Merkulova et al. (2014), openness was only significantly correlated with extraversion (*r* = *0*.34) and in our study it only correlated with agreeableness (*r* = 0.23). Thus, it is possible that the correlations between openness and perceived fairness observed by Truxillo et al. (2006) and Oostrom et al. (2010) were somewhat inflated due to the overlap between openness and the other personality traits in their measures.

Beyond the effective prediction of who will be a good employee, a selection procedure should not negatively affect applicants’ attraction to the job or organization (Ryan and Huth 2008). Contrary to concerns raised (Tran and Blackman 2006), our results showed that on average the applicants rated the group interview as both socially and structurally fair, with means above four on a five-point scale. This demonstrates that conducting group interviews that applicants experience as fair is possible.

### Strengths and Limitations

The present study examined how teacher job applicants reacted to an assessment of their views on students and learning, attitudes to innovation and the use of information technology, and cooperation and interaction skills (selection content) in a structured group interview (selection method). We did not employ a comparative design, for example, by studying whether group interviews would be evaluated as less fair if the content was different (e.g., focused on factual knowledge in a specific field of teaching) or by varying the method but keeping the content constant (e.g., individual versus group interviews). Because of this, we cannot ascertain to what extent the trait–fairness relationships observed in the present study are influenced by the specific content of the interviews or the group interview setting. A next potentially fruitful step in research on applicant personality and fairness reactions would be to systematically vary both selection content and methods so that general trait–justice relationships can be separated from content or method-specific relationships.

Another limitation is that we have not addressed how perceptions of fairness may result from the interaction between an applicant’s personality and the personality of the other applicants in the group. There is evidence that group personality composition affects both group and individual outcomes at work (Halfhill et al. 2005; Sung et al. 2014). Future research on the role of personality in perceptions of group-based selection tools should take this into account. It is also important to note that we cannot rule out that the screening prior to the interviews may have led to applicants with certain personality profiles to be more likely to be invited to the interviews than others.

Both the personality ratings (i.e., the predictors) and the fairness perceptions (i.e., the outcomes) come from the same source and method, the applicants’ questionnaire responses. This may introduce method bias into our results (Podsakoff et al. 2012), resulting in inflated or attenuated relationships between traits and fairness perceptions (Conway and Lance 2010). The fact that we measured personality and fairness at two different points in time (i.e., prior to the interview and after the interview, respectively) may have lessened the potential impact of method bias on our findings (Podsakoff et al. 2012). Moreover, we systematically compared the 60 items included in the NEO-FFI (Costa and McCrae 1992b; Martinsen et al. 2005) with the items included in our measure of social and structural fairness (Table 1) to explore whether the observed relationships could be artificially inflated due to wording similarities. Of the 60 items, we could identify only two which had wordings which resembled the wording of the fairness items.^1^ Others may validate this assertion by comparing our items (Table 1) with the items in the NEO-FFI.

Other researchers have pointed to the lack of research among applicants for permanent, full-time, and professional jobs (Hausknecht et al. 2004), to differences between the reactions of students and actual applicants (Chapman et al. 2005; Truxillo et al. 2009) and to differences in fairness reactions across lab and field settings (Truxillo et al. 2009). The respondents in our fields study were adult, highly educated, and actually applying for professional positions. This enhances our confidence in the generalizability of our findings to this type of applicant population.

When research on applicant personality and justice perceptions was last meta-analyzed (Hausknecht et al. 2004), the available evidence suggested that the relationships between personality traits and fairness perceptions were small. However, Hausknecht and colleagues could only meta-analytically investigate the effects of neuroticism and conscientiousness, because there were not enough primary studies including the other big five traits. The primary studies concerning the effect of conscientiousness and neuroticism had been conducted in hypothetical settings and did not include actual applicants (Hausknecht et al. 2004). Since then, more primary studies have been published. Some studies indicate that the relationships between applicant personality and fairness perceptions are small, but other studies (like ours) point to medium-sized effects. It is possible that the role of personality traits has been underestimated because trait interactions have been overlooked. Moreover, it is also possible that effects will vary with the type of selection tool that applicants react to. In order to know what the “true” relationships between applicant personality and fairness reactions are, we need enough studies with actual applicants reacting to different selection tools to include in meta-analyses. Our study contributes to this end.

### Implications

One reason for studying the relationships between applicant personality and fairness perceptions has been the concern that applicants with desirable traits (i.e., traits related to high job performance) will reject the job or organization due to negative reactions to the selection process (Truxillo et al. 2006). The results from the present study does not suggest that traits traditionally associated with higher job performance (e.g., conscientiousness and emotional stability; Barrick et al. 2001; Salgado 1997) are related to lower levels of perceived fairness of group interviews. More generally, we found that group interviews were on average perceived as high in fairness. These findings may be especially relevant for school leaders who are recruiting teachers. Both in Norway and in other countries, there is a shortage of qualified teachers; attrition rates from the profession are high and a substantial number of people with formal training as teachers chose to work in other sectors of the labor market (Skaalvik and Skaalvik 2011). Given this context, school leaders may be concerned with avoiding selection tools that are negatively perceived by qualified applicants. Our study gives no indications that school leaders or other HR-practitioners should avoid group interviews from a fairness perspective.

The results of this study demonstrated that the impact of applicant personality on procedural justice perceptions in selection may be underestimated if interactions between traits are not taken into consideration. Thus, future research in this area should include those trait interactions that are conceptually relevant to the selection tool applicants are faced with. For selection tools that involve extensive social interaction, like group selection interviews, researchers may want to consider interactions among neuroticism and extraversion and extraversion and agreeableness. For other selection tools, other interactions may be relevant.

## Footnotes

1 Specifically, this concerned two items from the agreeableness domain, both referring to one’s tendency to treat others with courtesy and consideration. These two items reflect the concept of consideration, respect, and honesty, which is mentioned in two of the items in the social justice scale (“I experienced the interviewer as honest and sincere” and “I was treated with consideration and respect during the group interview”). Importantly, however, the personality items refer to one’s own tendency to be courteous and considerate; the fairness items refer to the experience of being treated with courtesy and respect in a specific social situation.
